# Quantitative Airway Assessment of Diffuse Idiopathic Pulmonary Neuroendocrine Cell Hyperplasia (DIPNECH) on CT as a Novel Biomarker

**DOI:** 10.3390/diagnostics12123096

**Published:** 2022-12-08

**Authors:** Cormac O’Brien, John A. Duignan, Margaret Gleeson, Orla O’Carroll, Alessandro N. Franciosi, Dermot O’Toole, Aurelie Fabre, Rachel K. Crowley, Cormac McCarthy, Jonathan D. Dodd, David J. Murphy

**Affiliations:** 1 Department of Radiology, St. Vincent’s University Hospital, D04 T6F4 Dublin, Ireland; 2 National Centre for Neuroendocrine Tumours, ENETS NET Centre of Excellence, St. Vincent’s University Hospital, D04 T6F4 Dublin, Ireland; 3 Department of Respiratory Medicine, St. Vincent’s University Hospital, D04 T6F4 Dublin, Ireland; 4 School of Medicine, Trinity College Dublin, D02 R590 Dublin, Ireland; 5 Department of Pathology, St. Vincent’s University Hospital, D04 T6F4 Dublin, Ireland; 6 School of Medicine, University College Dublin, D04 V1W8 Dublin, Ireland; 7 Department of Endocrinology, St. Vincent’s University Hospital, D04 T6F4 Dublin, Ireland

**Keywords:** tomography, X-ray computed/methods, neuroendocrine tumor/diagnostic imaging, diffuse idiopathic neuroendocrine cell hyperplasia (DIPNECH), neuroendocrine tumors/pathology, lung neoplasms/pathology

## Abstract

Objectives: Diffuse idiopathic pulmonary neuroendocrine cell hyperplasia (DIPNECH) occurs due to abnormal proliferation of pulmonary neuroendocrine cells. We hypothesized that performing a quantitative analysis of airway features on chest CT may reveal differences to matched controls, which could ultimately help provide an imaging biomarker. Methods: A retrospective quantitative analysis of chest CTs in patients with DIPNECH and age matched controls was carried out using semi-automated post-processing software. Paired segmental airway and artery diameters were measured for each bronchopulmonary segment, and the airway:artery (AA) ratio, airway wall thickness:artery ratio (AWTA ratio) and wall area percentage (WAP) calculated. Nodule number, size, shape and location was recorded. Correlation between CT measurements and pulmonary function testing was performed. Results: 16 DIPNECH and 16 control subjects were analysed (all female, mean age 61.7 +/− 11.8 years), a combined total of 425 bronchopulmonary segments. The mean AwtA ratio, AA ratio and WAP for the DIPNECH group was 0.57, 1.18 and 68.8%, respectively, compared with 0.38, 1.03 and 58.3% in controls (*p* < 0.001, <0.001, 0.03, respectively). DIPNECH patients had more nodules than controls (22.4 +/− 32.6 vs. 3.6 +/− 3.6, *p* = 0.03). AA ratio correlated with FVC (R2 = 0.47, *p* = 0.02). A multivariable model incorporating nodule number, AA ratio and AWTA-ratio demonstrated good performance for discriminating DIPNECH and controls (AUC 0.971; 95% CI: 0.925–1.0). Conclusions: Quantitative CT airway analysis in patients with DIPNECH demonstrates increased airway wall thickness and airway:artery ratio compared to controls. Advances in knowledge: Quantitative CT measurement of airway wall thickening offers a potential imaging biomarker for treatment response.

## 1. Introduction

Diffuse idiopathic pulmonary neuroendocrine cell hyperplasia (DIPNECH) is a rare, underrecognized condition characterised by hyperplasia of Kulchitsky type pulmonary neuroendocrine cells [1]. Pulmonary neuroendocrine cells (PNEC) are specialised solitary epithelial cells that can proliferate with a pathological spectrum including neuroendocrine cell hyperplasia, tumorlets and carcinoid tumours [2]. PNEC hyperplasia is a proliferation of PNEC cells that does not cross the basement membrane [3] with the distinction between tumorlets and carcinoid tumours based on size >5 mm [4,5]. DIPNECH is associated with progression to carcinoid tumours and is categorised as a pre-invasive lesion for lung carcinoid tumours by the World Health Organization [5]. The pattern of involvement is usually either linear proliferation, multiple scattered proliferations, or small nodules [6]. Secondary bronchiolar chronic inflammation and fibrosis can be seen in association, ranging from peribronchial T cell infiltrate, bronchial wall thickening by fibrosis to constrictive bronchiolitis [2] corresponding with airway changes on CT.

DIPNECH detected incidentally in asymptomatic patients makes up ~50% of cases; when symptomatic it typically presents in the fifth or sixth decade of life and women are affected in >90% of cases [7]. The classic clinical presentation is a middle-aged female non-smoker with chronic cough and wheeze, mixed obstructive/restrictive pulmonary function tests (PFTs) and poor response to bronchodilators [4,8,9]. Symptomatic patients often have symptoms for a number of years, and are frequently misdiagnosed with asthma or chronic bronchitis [10]. DIPNECH poses a diagnostic challenge. In a recent report of 32 patients DIPNECH was clinically suspected at presentation in only one case and was mentioned by the interpreting radiologist in only 31% of CT reports [11]. 

**Figure 1:**
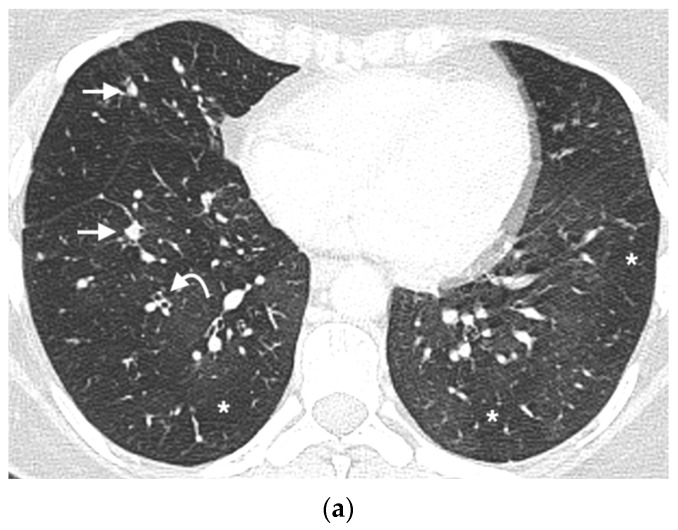

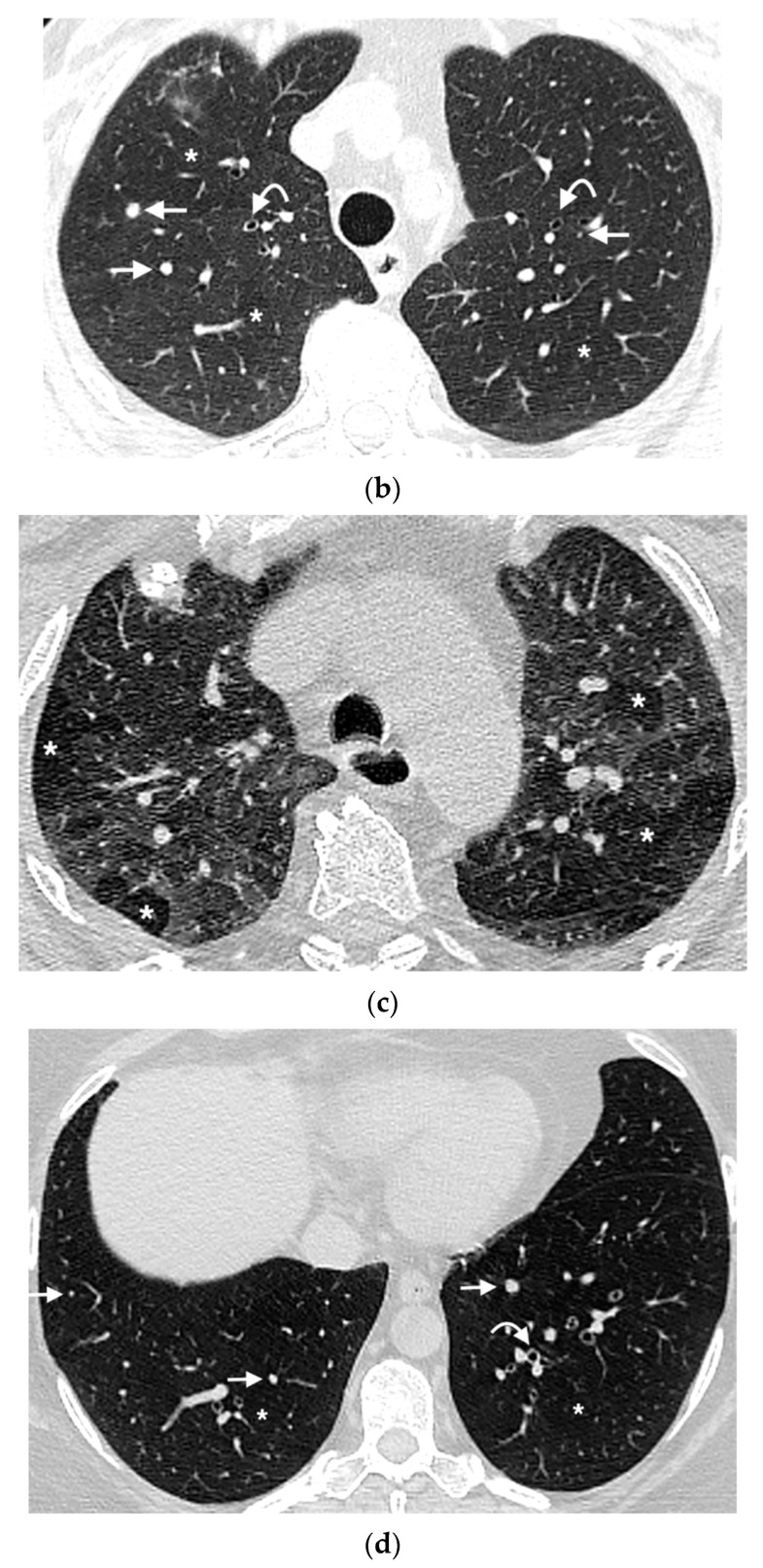
DIPNECH CT case examples. (**a**) Axial CT thorax in a middle aged woman with DIPNECH, status post right lung resection demonstrates multiple scattered small pulmonary nodules (arrows), bronchial wall thickening (curved arrow) and multifocal lobular and regional mosaic attenuation (*). (**b**) Axial CT thorax in an older woman with DIPNECH shows mild bronchial wall thickening (curved arrows), occasional scattered small pulmonary nodules (arrows) and mild lobular mosaic attenuation (*). (**c**) Expiratory CT image in the same patient demonstrates lobular and regional areas of air trapping (*). (**d**) Axial CT of the lung bases in a middle aged woman with DIPNECH showing bibasal pulmonary nodules (arrows), borderline dilated mildly thick-walled airways (curved arrow) and lobular mosaic attenuation (*).

The typical CT appearances of DIPNECH include mosaic attenuation [12] multiple pulmonary nodules and airway wall thickening, with bronchiectasis occasionally described (Figure 1) [13]. The potential role of quantitative CT in DIPNECH has not yet been assessed. Somatostatin analogue (SSA) therapy has been shown to be of benefit in some patients with DIPNECH [14], but predicting which patients will benefit is as yet unknown, with no objective markers of disease other than PFTs. We hypothesized that performing a quantitative analysis of airway features on chest CT may reveal differences to matched controls, which could ultimately help provide an imaging biomarker.

## 2. Materials and Methods

### 2.1. Patients

**Figure 2:**
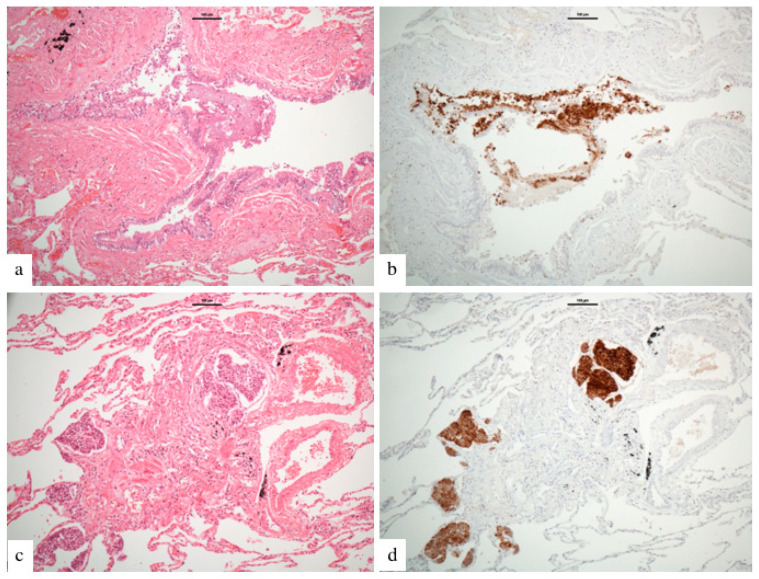
Histological features of DIPNECH in an adult female patient. (**a**) shows a bronchiole (hematoxylin and eosin stain) with (**b**) linear proliferation of neuroendocrine cells (brown immunostaining for chromogranin). (**c**) demonstrates a bronchiole (hematoxylin and eosin stain) with (**d**) multiple neuroendocrine tumourlets (brown immunostaining for chromogranin) (×100 magnification, scale bare 100 um).

Patients were retrospectively identified from St. Vincent’s University Hospital institutional databases. All patients that had an available CT thorax and a confirmed diagnosis of DIPNECH from 2011–2020 were included. The diagnosis of DIPNECH was based on combination of clinical, pathological and radiological features at the national neuroendocrine multidisciplinary team meeting at our institution, with pathology available in all cases (Figure 2 & Supplemental methods). A group of age and sex matched control subjects was selected by generating a random list of patients who had undergone CT thorax for lung nodule work-up from January–July 2020. Control subjects who had a background history of known active malignancy, diffuse lung disease or current infection were excluded. The local institutional ethics review board approved this retrospective review, and written informed consent was waived. Clinical information including basic patient demographics, clinical presentation and PFTs were collected.

### 2.2. CT Acquisition

All patients underwent volumetric 64-slice CT thorax with a mixture of CT acquisition protocols with and without IV contrast depending on the clinical indication (supplemental methods). Syngo Via post-processing software (Version VB60, Siemens Healthineers, Erlangen, Germany) was used for quantitative CT image analysis, which was performed randomly and blinded to the clinical information.

### 2.3. Quantitative Airway Measurement

**Figure 3:**
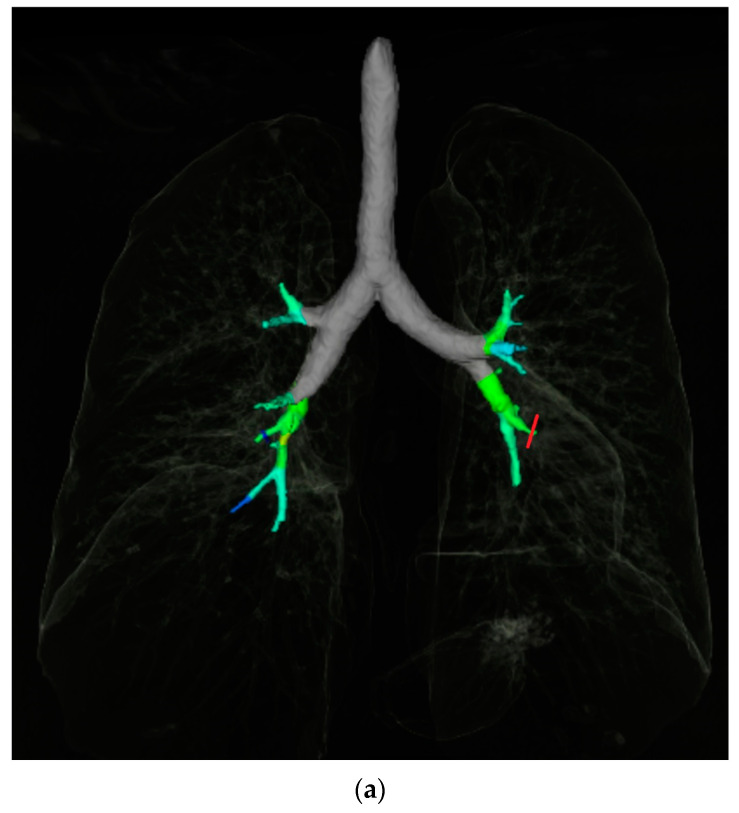

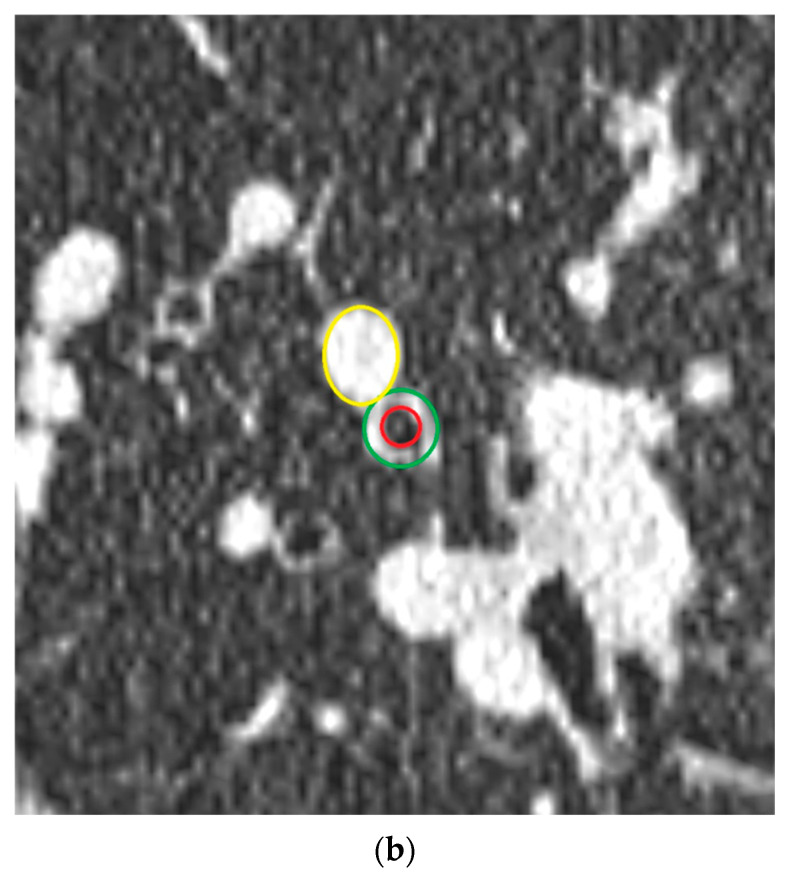
CT Airway measurement example. (**a**) is an example of segmented 3D volume rendered image of the bronchial tree generated from the CT data by SyngoVia image post processing software, with the segmental airway branches in various shades of green and blue. The red line signifies the bronchopulmonary segmental airway being measured in Figure 1b (left lower lobe lateral segment). (**b**) shows a corresponding multiplanar reformat image of the left lower lobe lateral segmental bronchus, double oblique to the centreline of the airway. Automated inner (red) and outer (green) airway area measurements are shown, along with measurement of the adjacent artery area (yellow).

Quantitative, automated airway analysis was performed using the “Pulmo 3D” workflow on Syngo Via software using previously validated techniques [15,16,17,18]. Using the volumetric CT data, the software automatically segments the airways, providing a 3D volume rendered map of the bronchial tree (Figure 3a). Segmental bronchi were categorised for the purpose of data collection according to the Boyden classification [19]. The centreline along the middle of the airway lumen was automatically delineated by the software, providing a navigable CT dataset of the airway and artery (AA) pair. Quantitative measurements of the segmental bronchi and their accompanying artery were performed for each segmental bronchus in all lobes. The reader manually selected the segmental bronchi for each lobe in turn, choosing an image plane as proximal as possible to the airway bifurcation that demonstrated the airway and corresponding artery. Quantitative measurements of airway outer and inner surface area were automatically generated by the software, and manually adjusted if needed. The accompanying artery surface area was measured quantitively using a circular region of interest (Figure 3b). In cases where the bronchial wall thickening was nodular or irregular, the narrowest airway segment was used. If the software was unable to segment a lobe or bronchopulmonary segment, no measurement was recorded. Segments where a parallel airway and artery could not be identified were also excluded.

### 2.4. Airway-Artery Pair Analysis

The quantitative airway-artery analysis was performed using previously validated techniques as follows. The diameters (d) for each AA pair were derived from the measured surface areas (a) using the following formula: d = 2 × √a∕π [18]. The AA ratio was calculated by dividing the outer airway diameter by the artery diameter.. The airway wall thickness (difference between outer and inner airway diameter) was divided by the artery diameter to compute the airway wall thickness:artery (AWTA) ratio [18]. The wall area percentage, a metric for luminal narrowing, was calculated by dividing the total airway area by the area occupied by its wall [15,16]. Wall area was calculated by subtracting inner airway area from outer airway area [20]. 

### 2.5. Mosaic Attenuation

Mosaic attenuation (MA) was graded using a previously validated 4-point qualitative scale (none, mild, moderate, severe) [11]. When present, the predominant pattern of mosaic attenuation was recorded as either lobular or regional.

### 2.6. Nodule Analysis

Automated computer aided detection (CAD) software was used for nodule identification using the “Multimodality (MM) reading” workflow on Syngo Via software (version VB60). Identified nodules were confirmed by a reviewing radiologist (COB, 5 years’ experience), who performed further nodule characterisation as follows: maximal size was measured with electronic calipers and their segmental position recorded. The total number of nodules in each bronchopulmonary segment was counted. Axial nodule location was characterised as either central (>1 cm from the pleural surface), peripheral (within 1 cm of the pleural surface) or subpleural (if any part of the nodule touched either the pleura or a fissure). Nodule shape was characterised as either spherical, ovoid, triangular or other [21]. If there was >1 nodule in a segment, the dominant shape for that segment was recorded. In lung segments with multiple nodules, the size of the largest and smallest nodule was recorded. CTs were also reviewed independently of the automated software to assess for any nodules not detected by the software. Distribution of nodules was characterised as either random, peribronchiolar or centrilobular [22]; this could only be performed in the DIPNECH group due the small number of nodules in controls.

### 2.7. Statistical Analysis

Statistical analysis was performed using SPSS (version 17.0, Chicago, IL, USA), GraphPad (GraphPad Software, San Diego, CA, USA) and R 4.0.4 (The R Foundation for Statistical Computing). Continuous variables are presented as mean ± standard deviation (SD). Categorical variables are presented as frequencies and percentages. Group comparison of continuous variables was performed by Student’s independent t test. Group comparisons of categorical variables were performed by Fisher’s exact test. Correlations were performed using Spearman’s rank test. Adjusted R2 was used to compare the models, with higher adjusted R2 equalling better fit. Receiver operating characteristic (ROC) curves with corresponding area under curves (AUC) were plotted to identify the quantitative CT metrics (nodule number, AA ratio, AWTA-ratio and WAP) with the highest sensitivity and specificity to differentiate DIPNECH patients from controls. Based on the observed univariate correlations we explored the performance of a multivariable model for discriminating DIPNECH and control CT features (supplemental methods). All statistical tests were 2-sided, and a value of *p* < 0.05 was considered significant.

## 3. Results

17 patients with tissue-confirmed DIPNECH were identified from our database, all female. One patient did not have a CT available for analysis and was excluded. There were 16 female control patients included, with no significant differences in age (61.2 +/− 10.8 vs. 61.5 +/− 13.4 years, respectively, *p* = 0.9) or smoking status (2 vs. 6, *p* = 0.2) between DIPNECH and controls. 13 had DIPNECH diagnosis confirmed at surgery, 3 with CT guided core lung biopsies.

### 3.1. Airway Wall Thickness and Airway Artery Ratios

In total, 425 segmental airway-artery (AA) pairs were measured between the two groups. 216 AA pairs (75% of total potential AA pairs, assuming conventional anatomy) were measured in the DIPNECH group and 209 (73%) in the controls (*p* = 0.53). The total AWTA-ratio was elevated in the DIPNECH patients compared to controls (0.57 +/− 0.42 vs. 0.380 + /− 0.23, respectively, *p* < 0.001) with significant differences for the right lung (0.61 +/− 0.52 vs. 0.39 +/− 0.30, respectively, *p* < 0.001), left lung (0.50 +/− 0.17 vs. 0.37 +/− 0.14, respectively, *p* < 0.001) and each individual lobe (Table 1). The AA ratio was significantly elevated for DIPNECH patients compared to controls for the lungs as a whole (1.18 +/− 0.53 vs. 1.03 +/− 0.29, respectively, *p* < 0.001), for the right lung (1.22 +/− 0.64 vs. 1.03 +/− 0.35, respectively, *p* = 0.007) and the left lung (1.10 +/− 0.27 vs. 1.00 +/− 0.20, respectively, *p* = 0.013). There was no correlation found between number of pulmonary nodules and either AWTA-ratio (R2= 0.02, *p* = 0.58) or AA ratio (R2= 0.02, *p* = 0.59).

### 3.2. Absolute Airway, Artery Measurements & Wall Area Percentage

There was no significant difference between the two groups in absolute airway outer diameter for the total lung (0.55 +/− 0.10 cm vs. 0.55 +/− 0.11 cm, *p* = 0.91), either lung or in any lobe (Table 2). The wall area percentage (WAP) was elevated in the DIPNECH patients compared to controls for the lungs as a whole (68.8 +/− 13.6% vs. 58.3 +/− 12.2%, respectively, *p* = 0.003), for the right lung (68.6 +/− 14.3% vs. 58.1 + /− 12.9%, *p* = 0.012) and for the left lung (67.9 +/− 15% vs. 57.4 +/− 12.2%, *p* < 0.001, Table 2). Airway inner diameter was significantly smaller in DIPNECH patients than controls (0.30 +/− 0.09 cm vs. 0.36 +/− 0.08 cm, *p* < 0.001), with the DIPNECH patients demonstrating increased absolute airway wall thickening (0.26 +/− 0.09 cm vs. 0.23 +/− 0.77 cm, *p* < 0.001). The paired artery diameter was smaller for the DIPNECH subjects than controls for the lungs as a whole (0.51 +/− 0.13 cm vs. 0.53 +/− 0.15, *p* = 0.01) and in each lung and individual lobe, although the latter did not all meet statistical significance (Table 2).

### 3.3. Mosaic Attenuation

Mosaic attenuation (MA) was present in all 16 DIPNECH CTs, compared with 4 (16%) of controls (*p* < 0.0001, Table 3). In the DIPNECH group, MA was scored as mild in 11 (69%) and moderate in 5 (31%) cases, with a lobular pattern of MA in 10 (53%) and regional MA in 6 (38%). Expiratory imaging was available in one DIPNECH patient, demonstrating air trapping. All control patients with MA had a mild, lobular pattern.

### 3.4. Nodule Characteristics

Patients with DIPNECH had more nodules compared to controls (average number 22.4 +/− 32.6 vs. 3.6 +/− 3.6, respectively, *p* = 0.03, Table 3). Amongst the DIPNECH cohort, the nodules were relatively evenly distributed between the upper and lower lobes (47% vs. 53%, respectively, *p* = 0.19), without a central or peripheral preponderance (36% vs. 35%, respectively, *p* = 0.88). DIPNECH nodules were most commonly spherical in shape (67%, *p* < 0.001), but this did not differ significantly compared to controls (59%, *p* = 0.35, Table 3). 47% of DIPNECH nodules had a predominately peribronchiolar distribution (Table 3). No DIPNECH nodules demonstrated calcification. The largest nodule in each lung segment ranged from 1.7–26 mm, with a median size of 7.1 mm (+/− 4.1) in the DIPNECH patient compared to 1–12.7 mm with a median size of 5.4 mm (+/− 2.6) in the control subjects (*p* = 0.005).

### 3.5. Clinical Markers

Amongst the DIPNECH group, ten patients (58%) were symptomatic, with 8 (47%) complaining of chronic cough, 7 (41%) with shortness of breath, two (11%) with wheeze and one patient describing symptoms of chest tightness. PFTs were available in eleven (64%) DIPNECH patients, with a mean forced expiratory volume in 1 s (FEV1) of 84.77 +/− 26.1% (range 42–127), forced vital capacity (FVC) of 101.54 +/− 25.9% (range 58–137) and diffusion capacity (DLCO) of 81.45 +/− 15.1% (range 57–104). There was a significant positive correlation between FVC and AA ratio (R2 = 0.47, *p* = 0.02). AWTA-ratio did not correlate with FVC (*p* = 0.09). We did not find any further significant correlation between either AA ratio or AWTA-ratio and FEV1 or DLCO. We did not find a significant difference between symptomatic and asymptomatic patients DIPNECH regarding the number of pulmonary nodules (27 +/− 40.6 vs. 14.8 +/− 10.4, respectively, *p* = 0.48), AA ratio (1.14 +/− 0.17 vs. 1.01 +/− 0.11, *p* = 0.15) or AWTA-ratio (0.53 +/− 0.17 vs. 0.48 +/− 0.12, *p* = 0.55). There was no significant correlation between the number of nodules and FEV1 (*p* = 0.25), FVC (*p* = 0.22), FEV1/FVC ratio (*p* = 0.88) or DLCO (*p* = 0.51).

### 3.6. Diagnostic Performance (Table 4)

**Figure 4:**
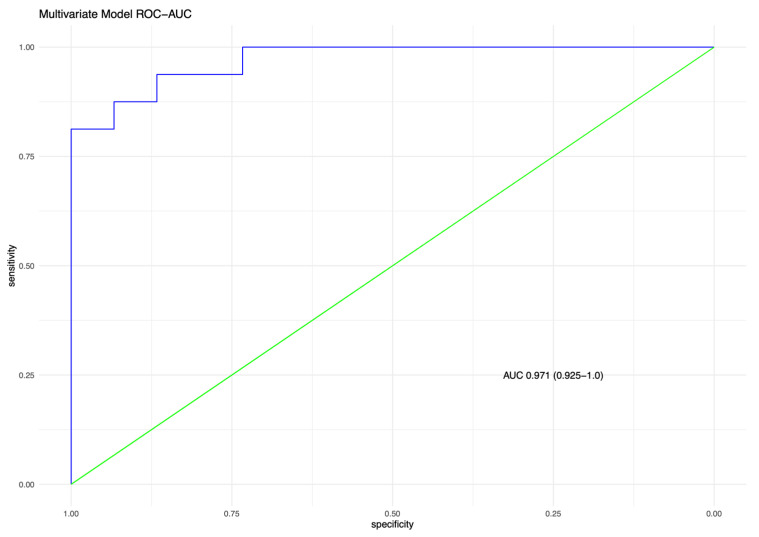
Receiver operating curves (ROC) for multivariate model for DIPNECH diagnosis. Combination of nodule number, Airway-artery ratio and Airway wall thickness-artery ratio (AUC 0.97).

For total nodule number, the ROC area under the curve (AUC) is 0.89, with ≥5 nodules the optimal cut-off point (sensitivity 0.81, specificity 0.87, Supplemental Figure S1A). For the AA ratio the AUC is 0.63, with an AA ratio cut-off ≥ 1.0 optimal (sensitivity 0.69, specificity 0.47, Supplemental Figure S1B). The AUC for the AWTA-ratio is 0.73, with ≥0.43 the best cut-off 0.43 (sensitivity 0.71, specificity 0.68, Supplemental Figure S1C). For WAP, ≥60% was the optimal cut off with an AUC of 0.73 (sensitivity 0.75, specificity 0.51, Supplemental Figure S1D). A multivariable model assessing the co-variates “nodule number”, “AA ratio” and “mean AWTA-ratio”, demonstrated a good overall performance for discriminating between DIPNECH and control CTs with an ROC-AUC of 0.97 (95% CI; 0.925–1.0 Figure 4). 

**Table 4:** Diagnostic performance of quantitative airway and nodule CT features for DIPNECH diagnosis.

CT Metric	Cut-Off	Sensitivity	Specificity	Positive LR	Negative LR
Nodule Number	≥5	0.81	0.87	6.2	0.2
Airway-artery ratio	≥1.0	0.69	0.47	1.3	0.6
Airway wall thickness-artery ratio	≥0.73	0.71	0.68	2.2	0.4
Wall Area Percentage (%)	≥60	0.75	0.51	1.5	0.5

DIPNECH = Diffuse idiopathic neuroendocrine cell hyperplasia; LR = Likelihood ratio.

## 4. Discussion

This is, to our knowledge, the first quantitative CT airway analysis in DIPNECH, and our results show that DIPNECH patients have significantly increased airway wall thickness and AA ratio than age and sex matched controls. There are several proposed explanations for the increased airway thickening in DIPNECH including excessive localised peptide secretion causing constrictive bronchiolitis with peribronchial, interstitial fibrosis [23], and direct local airway wall thickening from PNEC proliferation [3]. Somatostatin analogue (SSA) therapy has been shown to be of benefit in some patients with DIPNECH [14], but predicting which patients will benefit is as yet unknown- quantitative airway assessment could potentially provide an imaging biomarker to assess suitability for, and response to, treatment.

Previous studies describing bronchial wall thickening in DIPNECH have used qualitative assessments [24,25,26,27], with Little et al. [11] more recently performing a semi-quantitative assessment using a visual linear scale. We performed a fully quantitative airway assessment, based on methodology from other diffuse airways diseases, finding diffuse airway wall thickening. Studies examining quantitative chest CT analysis of airway wall changes in cystic fibrosis studies have proposed the AWTA-ratio as a robust measurement of airway inflammation, as it is indexed to the adjacent artery rather than relying on absolute measurements, with excellent inter-observer agreement [17,18,28]. COPD studies have proposed WAP measurement as a metric of airways disease [15].

One of the interesting results from our analysis is that DIPNECH patients had smaller mean absolute arterial diameter compared to controls. The AA ratio is based on outer airway diameter relative to the adjacent artery diameter, and has previously been validated as a more sensitive and objective method to detect bronchiectasis [28]. Bronchiectasis has been described as an infrequent feature of DIPNECH [29], with one series reporting it in 9% of cases based on a visual assessment [25]. Our results suggest that the observed increased AA ratio may not only reflect airway dilatation, but also underlying vasoconstriction. This relative vasoconstriction may be a manifestation of obstructive small airways disease; in addition we found a significant positive correlation between FVC and AA ratio, suggesting a correlation with air trapping, with all DIPNECH patients demonstrating mosaic attenuation. The excess neuroendocrine cell secreted peptides may have some direct effects on pulmonary vascular tone, which may also be contributory [30]. This interesting observation is worthy of further exploration, in particular to assess whether it is impacted by any therapeutic intervention.

As expected, DIPNECH patients had more nodules than controls. Multiple nodules have been reported in up to 49–81% of patients with DIPNECH [24,25,26]. Carr et al. [26] described innumerable (>50) nodules in ~1/3 of patients; 12.5% of our cohort met that criteria. Sazonova et al. previously proposed using a cut-off of >10 nodules in DIPNECH diagnostic criteria alongside age, female gender, the presence of symptoms and evidence of air trapping [24]. Our analysis suggest that fewer nodules (>5) are required to suggest a DIPNECH diagnosis. Unlike previous studies in which there was a preponderance of nodules in the lower lung zones [27], our analysis did not show any lobar preponderance. The majority of nodules in our DIPNECH cohort were spherical, and in a peribronchiolar distribution, similar to previous studies [26,27].

In practice, the presence of multiple small peribronchiolar nodules combined with lobular/regional mosaic attenuation in a middle aged/older woman are the most useful CT features when suggesting DIPNECH as a diagnosis. Our results suggest that a quantitative analysis of airway wall thickening and calibre provide an additional high degree of diagnostic accuracy in patients with multiple nodules, and potentially more importantly could serve as an imaging biomarker as therapeutic options advance.

Our study has several limitations. It is a retrospective, single centre study, the CT protocols included were not uniform, and expiratory imaging was only available in one case. The number of subjects included in this study is small as DIPNECH is a rare lung disease, but we performed the quantitative airway measurements at a bronchopulmonary segment level to adequately power the airway analysis. We used previously validated automated airway measurements, as this reduces the potential for human error [17,20]. The software used for airway analysis is based on attenuation values, and may be influenced by the presence of IV contrast-this is a potential limitation of our measurements, however we did not have a significant difference in the number of CTs performed with IV contrast between the two groups. Airway measurements were performed with one software only, and the generalisation of our results to measurements obtained with different software packages may be limited. The quantitative airway analysis performed in this study is currently too time consuming to be incorporated into routine clinical practice, but with the rapid advances in the use of artificial intelligence solutions in advanced post-processing applications, it is feasible that this type of quantitative analysis could be soon integrated into routine clinical workflow [31]. Our multivariable ROC analysis may be limited by overfitting on a small dataset, and may not reflect its true performance in real practice. Further quantitative analysis with larger cohorts may help create more objective diagnostic criteria and define imaging biomarkers.

## 5. Conclusions

Our quantitative CT analysis in patients with DIPNECH demonstrates increased airway wall thickening and airway:artery ratio compared to controls. The future incorporation of quantitative CT measurements of airway wall thickening and calibre into clinical radiology practice could potentially function as an imaging biomarker to assess suitability for, and response to, treatment such as somatostatin analogue therapy.

## Figures and Tables

**Table 1:** Airway wall thickness artery & airway-arterial ratios.

	DIPNECH (*n* = 216)	Control (*n* = 209)	*p* Value
**Airway wall thickness-artery ratio**			
Total lung	0.57 +/− 0.42	0.38 +/− 0.23	<0.001
Right lung	0.61 +/− 0.52	0.39 +/− 0.30	<0.001
Right upper lobe	0.56 +/− 0.30	0.37 +/− 0.33	0.0057
Right middle lobe	0.56 +/− 0.26	0.33 +/− 0.11	<0.001
Right lower lobe	0.65 +/− 0.68	0.41 +/− 0.27	0.01
Left lung	0.50 +/− 0.17	0.37 +/− 0.14	<0.001
Left upper lobe	0.52 +/− 0.14	0.41 +/− 0.18	0.002
Left lower lobe	0.49 +/− 0.20	0.35 +/− 0.12	<0.001
**Airway-artery ratio**			
Total lung	1.18 +/− 0.53	1.03 +/− 0.29	<0.001
Right lung	1.22 +/− 0.64	1.03 +/− 0.35	0.007
Right upper lobe	1.23 +/− 0.43	1.09 +/− 0.43	0.124
Right middle lobe	1.24 +/− 0.46	0.93 +/− 0.16	0.004
Right lower lobe	1.21 +/− 0.49	0.99 +/− 0.30	0.045
Left lung	1.10 +/− 0.27	1.00 +/− 0.20	0.013
Left upper lobe	1.13 +/− 0.33	1.06 +/− 0.30	0.298
Left lower lobe	1.07 +/− 0.21	1.05 +/− 0.40	0.78

Results are presented as mean +/− standard deviation, unless otherwise specified; DIPNECH = Diffuse idiopathic neuroendocrine cell hyperplasia.

**Table 2:** Airway and artery diameters, wall area percentage.

	DIPNECH (*n* = 216)	Control (*n* = 209)	*p* Value
**Airway outer diameter (cm)**			
Total lung	0.55 +/− 0.10	0.55 +/− 0.11	0.91
Right lung	0.55 +/− 0.10	0.54 +/− 0.12	0.37
Right upper lobe	0.54 +/− 0.09	0.57 +/− 0.14	0.19
Right middle lobe	0.56 +/− 0.17	0.56 +/− 0.08	0.92
Right lower lobe	0.55 +/− 0.09	0.50 +/− 0.15	0.04
Left lung	0.55 +/− 0.09	0.57 +/− 0.09	0.33
Left upper lobe	0.56 +/− 0.07	0.57 +/− 0.79	0.62
Left lower lobe	0.55 +/− 0.12	0.57 +/− 0.10	0.37
**Airway inner diameter (cm)**			
Total lung	0.30 +/− 0.09	0.36 +/− 0.08	<0.001
Right lung	0.30 +/− 0.09	0.361+/− 0.09	<0.001
Right upper lobe	0.30 +/− 0.08	0.38 +/− 0.12	0.0013
Right middle lobe	0.30+/− 0.11	0.36 +/− 0.07	0.0156
Right lower lobe	0.30 +/− 0.09	0.35 +/− 0.07	0.0031
Left lung	0.30 +/− 0.09	0.36 +/− 0.09	<0.001
Left upper lobe	0.31 +/− 0.09	0.34 +/− 0.08	0.027
Left lower lobe	0.29 +/− 0.09	0.38 +/− 0.10	<0.001
**Airway wall thickness (cm)**			
Total	0.26 +/− 0.09	0.22 +/− 0.08	<0.001
Right lung	0.25 +/− 0.07	0.20 +/− 0.06	<0.001
Right upper lobe	0.24 +/− 0.06	0.12 +/− 0.06	0.01
Right middle lobe	0.26 +/− 0.85	0.20 +/− 0.06	0.006
Right lower lobe	0.25 +/− 0.07	0.21 +/− 0.49	0.006
Left lung	0.28 +/− 0.11	0.23 +/− 0.09	0.004
Left upper lobe	0.32 +/− 0.114	0.27 +/− 0.11	0.051
Left lower lobe	0.25 +/− 0.01	0.19 +/− 0.05	0.002
**Wall Area Percentage (%)**			
Total	68.8 +/− 13.6	58.3 +/− 12.2	0.003
Right lung	68.6 +/− 14.3	58.1 +/− 12.9	0.012
Right upper lobe	68.1 +/− 12.3	56.8 +/− 15.5	<0.001
Right middle lobe	66.5 +/− 18.6	53.1 +/− 16.5	0.03
Right lower lobe	68.8 +/− 14.8	60.6 +/− 9.0	<0.001
Left lung	67.9 +/− 15.0	57.4 +/− 12.2	<0.001
Left upper lobe	69.4 +/− 13.5	59.5 +/− 13	<0.001
Left lower lobe	66.5 +/− 16.2	55.3 +/− 11	<0.001
**Artery diameter (cm)**			
Total lung	0.51 +/− 0.13	0.54 +/− 0.15	0.01
Right lung	0.49 +/− 0.13	0.52 +/− 0.17	0.09
Right upper lobe	0.47 +/− 0.14	0.56 +/− 0.15	0.007
Right middle lobe	0.46 +/− 0.08	0.61 +/− 0.09	<0.001
Right lower lobe	0.51 +/− 0.13	0.46 +/− 0.19	0.09
Left lung	0.52 +/− 0.14	0.56+/− 0.10	0.02
Left upper lobe	0.52 +/− 0.12	0.55 +/− 0.10	0.3
Left lower lobe	0.51 +/− 0.15	0.57 +/− 0.11	0.03

Results are presented as mean +/− standard deviation, unless otherwise specified; DIPNECH = Diffuse idiopathic neuroendocrine cell hyperplasia.

**Table 3:** Nodule Characteristics & Mosaic Attenuation.

	DIPNECH (*n* = 16)	Controls (*n* = 16)	*p* Value
**Nodule number**			
Total Number	22.4 +/− 32.6	3.6 +/− 3.6	0.03
Right lung	4.2 +/− 6.8	0.6 +/− 1.0	<0.001
Right upper lobe	4.3 +/− 7.3	0.7 +/− 0.9	0.059
Right middle lobe	2.1 +/− 2.2	0.4 +/− 0.7	0.006
Right lower lobe	6.3 +/− 8.6	0.8 +/− 1.4	0.018
Left lung	2.8 +/− 5.2	0.5 +/− 0.8	<0.001
Left upper lobe	2.1 +/− 2.9	0.3 +/− 0.6	0.002
Left lower lobe	4.9 +/− 8.1	0.8 +/− 1.1	0.053
**Nodule Location (%)**			
Peripheral	36	41	0.5
Central	35	37	0.7
Perifissural	12	16	0.47
Subpleural	18	6	0.06
**Nodule shape (%)**			
Spherical	67	59	0.35
Ovoid	22	22	1.0
Triangular	10	20	0.3
Other	1	0	1.0
**Nodule Distribution (%)**			
Centrilobular	20	-	
Peribronchiolar	47	-	
Random	33	-	
**Largest Nodule Diameter (mm)**			
Total Lung	7.1 +/− 4.1	5.4 +/− 2.6	0.005
Right Lung	7.2 +/− 4.9	5.56 +/− 2.7	0.048
Right upper lobe	6.1 +/− 2.4	4.8 +/− 3	0.11
Right middle lobe	8.5 +/− 6.2	5.4 +/− 1.4	0.09
Right lower lobe	7.3 +/− 4.4	6.3 +/− 3.1	0.27
Left Lung	7 +/− 3.4	5.3 +/− 2.6	0.02
Left upper lobe	6.9 +/− 2.8	5.8 +/− 2	0.16
Left lower lobe	7.1 +/− 3.9	4.8 +/− 2.4	0.03
**Mosaic attenuation**			
Overall	16	4	<0.0001
Mild	11	4	0.035
Moderate	5	0	0.04
Severe	0	0	-

Results are presented as mean +/− standard deviation, unless otherwise specified; DIPNECH = Diffuse idiopathic neuroendocrine cell hyperplasia.

## Data Availability

The data presented in this study are available on request from the corresponding author.

## Supplementary Materials

The following are available online at https://www.mdpi.com/article/10.3390/diagnostics12123096/s1, Supplemental Figure S1: Receiver operating curves (ROC) of quantitative CT metrics for the diagnosis of DIPNECH; Supplemental Figure S1A: ROC for Nodule number in DIPNECH diagnosis (AUC 0.89); Supplemental Figure S1B: ROC for Airway-artery ratio in DIPNECH diagnosis (AUC 0.63); Supplemental Figure S1C: ROC for Airway wall thickness-artery ratio in DIPNECH diagnosis (AUC 0.73); Supplemental Figure S1D: ROC for Wall area percentage thickening in DIPNECH diagnosis (AUC 0.73); Supplemental Methods. References [32,33] are cited in the Supplementary Materials.

## Abbreviations

AA ratio	Airway:artery ratio
AWTA ratio	Airway wall thickness:artery ratio
AUC	Area under the curve
DIPNECH	Diffuse idiopathic pulmonary neuroendocrine cell hyperplasia
CAD	Computer aided detection
CF	Cystic fibrosis
COPD	Chronic obstructive pulmonary disease
DLCO	Diffusion capacity
FEV1	Forced expiratory volume in 1 second
FVC	Forced vital capacity
LR	Likelihood ratio
MA	Mosaic attenuation
PFT	Pulmonary function test
PNEC	Pulmonary neuroendocrine cells
ROC	Receiver operating curve
SSA	Somatostatin analogue
WAP	Wall area percentage
