# Supplementary material for: Quantitative Airway Assessment of Diffuse Idiopathic Pulmonary Neuroendocrine Cell Hyperplasia (DIPNECH) on CT as a Novel Biomarker
Source: Diagnostics (Basel). 2022 Dec 8;12(12):3096. doi: 10.3390/diagnostics12123096 (PMC9776594; doi:10.3390/diagnostics12123096)
Supplement: Supplementary file 1 [file diagnostics-12-03096-s001.zip › DJM_Supplemental file 1 Methods.docx]

**Supplemental methods**

**Pathology**

For patients with surgical specimens available, the proposed minimum set of pathologic criteria to diagnose DIPNECH was applied, with at least multifocal neuroendocrine cells hyperplasia along small bronchioles combined with 3 or more carcinoid tumorlets [32]. For patients with core biopsies, we used a tissue diagnosis of carcinoid tumour along with typical CT appearances.

**CT acquisition**

All CT images were acquired on a 64-slice single-source CT system (Siemens Sensation 64, Siemens Medical Solutions, Forchheim, Germany). A variety of chest CT protocols including CT pulmonary angiography (CTPA), standard arterial phase chest CT (including as part of a CT chest, abdomen and pelvis oncology protocol) and non-contrast chest CT (with or without expiratory imaging) were included in this retrospective analysis. Slice thickness was </=1mm, slice interval was 0.5, kVp was 120, mA was between 100-350. Where contrast was given, a volume of between 50-75mls iodinated contrast (Omnipaque 350, GE Healthcare, Oslo, Norway) was injected using an infusion pump at a rate between 3-4mls/second followed by a saline chaser (20mls). All scans were breath-hold inspiration scans and performed without ECG-gating. In the DIPNECH group, 10 patients had an arterial phase chest CT with IV contrast, one patient had a CTPA and 5 patients had non-contrast chest CT, one of these with expiratory imaging. In the control group, 13 patients had arterial phase chest CT with IV contrast and 3 had a non-contrast CT. No patients in the control group had expiratory phase imaging.

**Statistical Analysis**

Based on the observed univariate correlations between DIPNECH and nodule number, airway-artery ratio, airway wall thickness-artery ratio and wall area percentage thickening, we explored the performance of a multivariable model for discriminating DIPNECH and control CT features. Mean airway-artery ratio, airway wall thickness-artery ratio and wall area percentage thickening were calculated for each subject (i.e. the subject mean of the individual lobar segment values). Generalised binomial logistic regression models were fitted (*glm* in R), with outcome set as DIPNECH (vs control) and nodule number, airway-artery ratio, airway wall thickness- artery ratio and wall area percentage thickening as co-variates. To minimise overfitting, various models were assessed by serial removal and replacement of the co-variates. The best model was determined using the *compare.model* function in R, based on the Aikake information Criteria (AIC) and Bayesian Information Criteria (BIC). AUC-ROC curves and 95% CI were generated using the pROC package in R[33].

**References**

32 Marchevsky AM, Wirtschafter E, Walts AE. The spectrum of changes in adults with multifocal pulmonary neuroendocrine proliferations: what is the minimum set of pathologic criteria to diagnose DIPNECH? Hum Pathol 2015;2:176-81. doi: 10.1016/j.humpath.2014.10.019

33 Robin X, Turck N, Hainard A, et al. pROC: an open-source package for R and S+ to analyze and compare ROC curves. BMC Bioinformatics. 2011;1:77. doi:10.1186/1471-2105-12-77
