# Supplementary material for: Quantitative Airway Assessment of Diffuse Idiopathic Pulmonary Neuroendocrine Cell Hyperplasia (DIPNECH) on CT as a Novel Biomarker
Source: Diagnostics (Basel). 2022 Dec 8;12(12):3096. doi: 10.3390/diagnostics12123096 (PMC9776594; doi:10.3390/diagnostics12123096)

**Supplemental Figures**

**Supplemental Figure S1- Receiver operating curves** (**ROC) of quantitative CT metrics for the diagnosis of DIPNECH**

**Supplemental Fig S1A**. ROC for Nodule number in DIPNECH diagnosis (AUC 0.89).


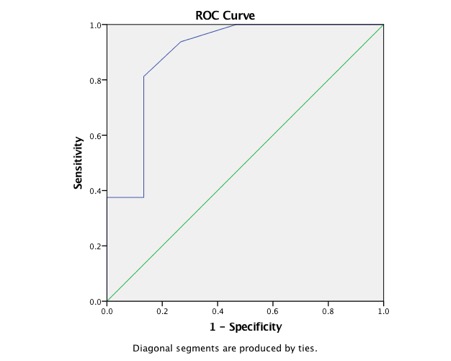


**Supplemental Fig S1B**. ROC for Airway-artery ratio in DIPNECH diagnosis (AUC 0.63).


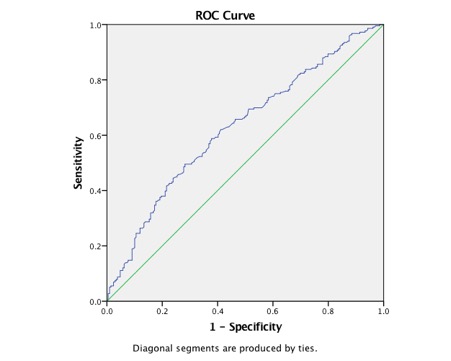


**Supplemental Fig S1C**. ROC for Airway wall thickness- artery ratio in DIPNECH diagnosis (AUC 0.73).
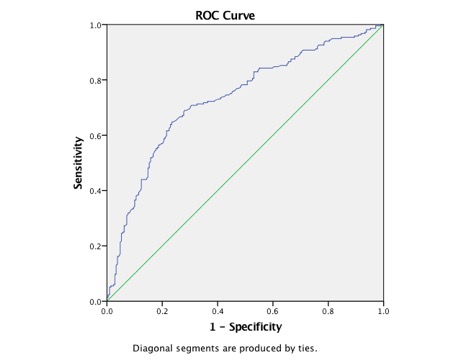


**Supplemental Fig S1D**. ROC for Wall area percentage thickening in DIPNECH diagnosis (AUC 0.73).


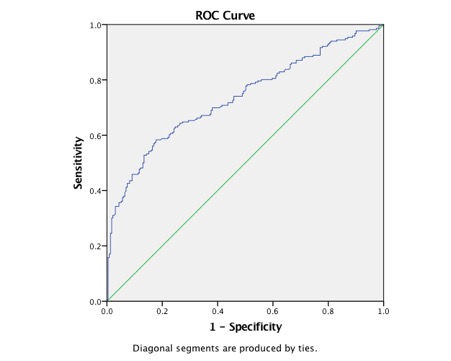

Supplement: Supplementary file 1 [file diagnostics-12-03096-s001.zip › Supplemental file 2-Figures.docx]
